# Twelve years with a capitation payment system in Swedish dental care: longitudinal development of oral health

**DOI:** 10.1186/s12903-021-01463-w

**Published:** 2021-03-06

**Authors:** Charlotte Andrén Andås, Magnus Hakeberg

**Affiliations:** 1 Public Dental Service, PO Box 7163, 40233 Gothenburg, Region Västra Götaland Sweden; 2 Department of Behavioral and Community Dentistry, Institute of Odontology, Sahlgrenska Academy, University of Gothenburg, Gothenburg, Sweden

**Keywords:** Capitation, Dental caries, Fee-for-service, Oral health

## Abstract

**Background:**

Since 2007, patients receiving oral health care within the Public Dental Service in Sweden have had the possibility to choose between the traditional fee-for-service (FFS) payment system or the new capitation payment system, ‘Dental Care for Health’ (DCH). Payment models are believed to involve different incentive structures for patients and caregivers. In theory, different incentives may lead to differences in health-related outcomes, and the research has been inconclusive. This 12-year longitudinal prospective cohort study of patients in regular dental care analyzes oral health development and self-reported oral health in relation to the patients’ level of education in the two payment systems, and compares with the results from an earlier 6-year follow-up.

**Methods:**

Information was obtained through a questionnaire and from a register from n = 5877 individuals who kept their original choice of payment model for 12 years, 1650 patients in DCH and 4227 in FFS, in the Public Dental Service in Region Västra Götaland, Sweden. The data comprised manifest caries prevalence, levels of self-reported oral health and education, and choice of dental care payment model. Analyses were performed with chi square and multivariable regression analysis.

**Results:**

The findings from the 6-year follow-up were essentially maintained at the 12-year examination, showing that the pre-baseline caries prevalence is the most influential factor for less favorable oral health development in terms of the resulting caries prevalence. Educational level (≥ university) showed an increased influence on the risk of higher caries prevalence after 12 years and differed between payment models with regard to the relation to self-rated oral health.

**Conclusions:**

Differences in health and health-influencing properties between payment models were sustained from 6 to 12 years. Strategies for making use of potential compensatory mechanisms within the capitation payment system to increase oral health equality should be considered.

## Background

‘Frisktandvård’ (“Dental Care for Health”, DCH) is a capitation payment option that was introduced in all Public Dental Service (PDS) organizations in Sweden in 2009, in parallel with the existing fee-for-service (FFS) system. The new payment scheme has thus been optional for the last 12 years, for individuals over 20 years of age who receive their dental health care within the Public Dental Service in Sweden. Nearly half of the adult population in Sweden uses the PDS and can choose between the two payment schemes. The rest of the adult population see private practitioners, at present without a capitation option other than exceptionally.

The DCH scheme involves monthly payments, determined in advance according to risk, for all basic dental care needed, provided that a self-care program is accepted and adhered to. The terms are renegotiated every 3 years, and have been described in greater detail elsewhere [1]. Today, around 792,000 individuals have chosen to pay according to DCH in Sweden and have been placed in ten different risk categories/fee classes. In Region Västra Götaland (VGR), the capitation scheme was introduced already in 2007. The VGR is situated in the south-western part of Sweden and has approximately 1.7 million inhabitants, corresponding to 17% of the total Swedish population. Here, DCH covers some 230,000  individuals, from 24 to above 90 years of age, and accounts for 48% of all patients attending the PDS. Twenty-nine per cent of all DCH payers in Sweden reside in the VGR.

FFS, on the other hand, is the default system that still operates on a payment per item basis, with an individually determined recall plan based on the patient’s risk of oral ill health. The cost of each item of dental care is determined by the current PDS price list, and is included in the National Dental Insurance (NDI) for all Swedish citizens. One of the essential features of the NDI is a cumulative high-cost protection scheme during a maximum treatment period of 1 year.

The original reason for offering a contract-based dental payment system was to enhance the preventative approach to oral care, thereby reducing the number of necessary restorative interventions, and ultimately to improve oral health [2].

Research into payment schemes in dentistry indicates a variety of effects, depending on the terms of the schemes and the parties involved, which, in turn, are related to how dental care is organized nationally. In conclusion, both overtreatment and undertreatment effects can be expected, and the net effect for the individual is highly dependent on other individual properties [3–12]. For DCH in the VGR, several papers [1, 13–18] show the following, in brief: Individuals who chose the capitation arrangement differed from those continuing with the FFS system by being younger, expressing greater interest in health-promoting actions, and considering oral health to be more important for their wellbeing. They also had a more favorable household economy. Treatment in DCH included more preventative care and fewer restorative efforts. Individuals in DCH were more content with their choice of payment scheme but not fully aware of the terms. After 6 years in either system, the risk of caries was 1.5 times higher in FFS, when prior caries experience and other important background factors were controlled for. Caregivers, on the other hand, experienced a loyalty conflict brought on by the diversification of payment options, in terms of becoming an insurance agent.

Continued long-term follow-up is needed to investigate expected as well as unexpected consequences when care conditions are modified. Longitudinal investigations may be even more important when large groups of people are involved, and effects may be amplified by covariation with other individual properties, such as sex, age, education, or indicators of health status.

The aim of this study was thus to compare individuals in the two payment schemes with regard to caries, and self-reported oral health relative to the level of education, and to extend the follow-up time from the earlier 6 years [13] to 12 years.

## Methods

The 5877 individuals included in this study constituted a subset of more than 13,000 regular PDS patients, introduced on the first possible occasion to the choice between the FFS system and the new capitation payment scheme DCH in VGR, Sweden, in 2007 [1]. This study’s subset represented those who had kept their original payment scheme since their first choice, 4227 patients in FFS and 1650 in DCH. These 5877 individuals also had a reported treatment time of ≥ 30 min per year. All individuals were classified according to their risk of development of oral ill health after each dental examination, which was scheduled according to the patient’s individual recall plan. The group allocation was determined by the caregiver on the basis of a suggested level from the digital risk classification tool R2, which, in turn, included both automatically retrieved data from patient files and manually completed information. R2 was used for risk assessment in VGR and in several other regional PDS organizations, however, no scientific evaluation has been published. The resulting risk category was based on oral disease and restoration status, modified by several individual risk-related factors, anamnestic as well as clinically collected; for example, diet, fluoride use, tobacco, oral hygiene, medical risk, and a self-assessment of oral health. All digitally retrieved information was reviewed and, if necessary, modified, at separate parameter level, as well as at the resulting 1–10 classification level. For those choosing DCH, the risk classification level (1–10) constituted the payment scheme fee class.

Register data from standard care, including information on the choice of payment model (DCH or FFS), age, prevalence of caries pre baseline and after 6 and 12 years, and self-rated level of oral health, was obtained from the T4 patient file system (T4 Practice Management Software, Carestream Dental, Stockholm, Sweden). Data from the original questionnaire in 2007 provided information on individual educational levels. All procedures were repeated as described earlier [13].

Caries was defined as manifest dentin caries (D3) or secondary caries (S). The number of caries lesions was trichotomized into 0/1–2/≥ 3. Educational level was dichotomized into university level (≥ 13 years) vs. below university level (≤ 12 years). Self-rated oral health was dichotomized into good (very good + good) versus poor (moderate + low).

Covariation between dependent and independent variables was analyzed with χ^2^ analysis. For the regression analysis, negative binomial modeling was chosen for over-dispersed non-normally distributed count data, with the number of manifest caries lesions after 12 years in the same payment model as the dependent variable. The independent variables included the number of baseline caries lesions, age, sex, educational level and payment model.

Data were handled and calculations performed in IBM SPSS Statistics, version 26. Ethical approval was obtained from the Swedish Ethical Review Authority, registration no. 323-07. All methods were performed in accordance with the relevant guidelines and regulations.

## Results

The number of individuals who had stayed with the same payment model during the 12 years from the implementation of DCH was 5877, a decrease by 422 individuals compared with 6299 at the 6-year follow-up (Table 1). The share of DCH payers after 6 years was 26.6% and 28% after 12 years. Since the start in 2007, 52% of the individuals who chose DCH were still contract holders in 2018. Forty-seven per cent of the original FFS payers still attended the PDS for their regular dental care (data not shown).

**Table 1:** Baseline variables by payment system and in the total sample, at six and twelve years

	N (%)	Age x̄^1^	Sex %		Education %		Pre-baseline caries % with lesions		
		years	*♀*	*♂*	≥13 years	≤ 12 years	0	1–2	≥ 3
*All*									
6 years	6299	44	53.2	46.8	33.0	67.0	65.7	25.8	8.5
12 years	5877	43.7	50.5	49.5	30.6	69.4	65.7	25.5	8.8
*DCH*^*2*^									
6 years	1675 (26.6)	36.3	53.0	47.0	30.9	69.1	71.9	23.6	4.5
12 years	1650 (28.1)	35.5	57.3	42.7	36.1	63.9	71.6	23.8	4.7
*FFS*^*3*^									
6 years	4624 (73.4)	46.8	53.2	46.8	33.7	66.3	63.5	26.6	9.9
12 years	4227 (71.9)	46.8	47.9	52.1	28.5	71.5	63.4	26.2	10.4

^1^At baseline

^2^New capitation payment system, ‘Dental Care for Health’

^3^Traditional fee-for-service payment system

The sex difference between the payment models increased between the 6 and 12-year follow-ups, with an increased female share in DCH, to a statistically significant difference between the payment models. At 12 years, the share of individuals with university level education had decreased marginally in the total sample. The trend differed between the two payment systems, since educational level increased in DCH and decreased in FFS, so that the educational level in DCH went from statistically significantly lower to higher than FFS (Table 1).

The distribution of caries, expressed as percentages in the categories with 0, 1–2 or ≥ 3 manifest lesions, differed statistically significantly between the payment models at both follow-ups, as well as at the pre-baseline registration (Table 2). The number of caries lesions showed a continuous decline from pre-baseline to the 6-year follow-up and further to the 12-year follow-up. All described differences in DCH and FFS between the registrations at 6 and 12 years were statistically significant, however not described in tables.

**Table 2:** Percentage of individuals per category with 0, 1–2 or ≥ 3 manifest caries lesions^1^, per payment model, pre-baseline, at 6 and 12 years

	No. of manifest caries lesions^1^ (%)			*p*^2^
	0	1–2	≥ 3	
*Pre-baseline*				
All	65.7	25.5	8.8	
DCH	71.6	23.8	4.7	< 0.001
FFS	63.4	26.2	10.4	
*6 years*				
All	73.1	21.5	5.4	
DCH	76.6	20.1	3.3	< 0.001
FFS	71.9	22.0	6.1	
*12 years*				
All	81.6	15.1	3.4	
DCH	82.2	16.0	1.8	< 0.001
FFS	81.3	14.7	4.0	

^1^D3 + S

^2^χ^2^

Patients rated their own oral health differently in the two payment models, after 6 and 12 years, respectively, and compared with one another: “poor” in about 20% of FFS but 5% in DCH, and “good” in about 80% in FFS but 95% in DCH. When grouped by educational level, the difference between the “poor” and “good” percentages was statistically significant for FFS, but not for DCH (Table 3).

**Table 3:** Percentage of individuals in each payment model with good or poor self-rated oral health at 6 years and 12 years, and by educational level

Self-rated oral health	DCH			FFS		
	Good %	Poor %	*p*	Good %	Poor %	*p*
Education years						
*6 years*						
All	96.2	3.8		80.9	19.5	< 0.001^1^
≥ 13	96.4	3.6	0.873^2^	86.1	13.9	< 0.001^2^
< 12	96.1	3.9		78.5	21.5	
*12 years*						
All	95.7	4.1		80.2	18.9	< 0.001^1^
≥ 13	96.4	3.6	0.463^2^	87.5	12.5	< 0.001^2^
≤ 12	95.3	4.4		77.4	21.4	

^1^ χ^2^. Between payment systems

^2^ χ^2^. Between educational levels, within payment system

The incidence rate ratio for manifest caries after 12 years showed a similar pattern to after six years. The most influential variable was, still, the pre-baseline caries prevalence, with an IRR of 2.42 (95% CI 2.08–2.82) (compared with 2.63 (2.31–3.00) at 6 years)) if ≥ 3 lesions prior to study start, and 1.33 (1.18–1.50) (1.40 (1.27–1.54) at 6 years)), if 1–2 lesions. The influence of the payment method was slightly reduced, with IRR = 1.51 (1.35–1.69) at 6 years to 1.39 (1.22–1.59) from 6 to 12 years. However, at 12 years, education ≤ 12 years seemed to increase the risk 1.36 (1.20–1.53) times instead of an earlier 1.12 times (1.02–1.23). Moreover, from no significance at 6 years, male sex was now seen to significantly increase the risk of manifest caries with IRR = 1.24 (1.11–1.38) (Table 4).

**Table 4:** Negative binomial regression analysis describing the influence of covariates on manifest caries incidence at the 6 and 12-year follow-ups, respectively

	B		SE		IRR^1^		CI (95%)		*p*	
	6 years	12 years	6 years	12 years	6 years	12 years	6 years	12 years	6 years	12 years
*Constant*	− 0.87	− 1.43	0.093	0.114	0.42	0.24	0.35–0.50	0.19–0.30	< 0.001	< 0.001
*Payment system choice*										
DCH^2^ *(Ref.)*					1	1				
FFS^3^	0.41	0.33	0.57	0.69	1.51	1.39	1.35–1.69	1.22–1.59	< 0.001	< 0.001
*Pre-baseline caries incidence*										
0 lesions *(Ref.)*					1	1				
1–2 lesions	0.34	0.29	0.050	0.061	1.40	1.33	1.27–1.54	1.18–1.50	< 0.001	< 0.001
≥ 3 lesions	0.97	0.88	0.067	0.078	2.63	2.42	2.31–3.00	2.08–2.82	< 0.001	< 0.001
*Age*										
years	− 0.01	− 0.01	0.002	0.002	0.99	0.99	0.98–0.99	0.99–0.99	< 0.001	< 0.001
*Education*										
≥ 13 years . *(Ref.)*					1	1				
≤ 12 years	0.11	0.31	0.048	0.062	1.12	1.36	1.02–1.23	1.20–1.53	0.021	< 0.001
*Gender*										
♀ *(Ref)*					1	1				
♂	− 0.02	0.21	0.044	0.054	0.98	1.24	0.90–1.07	1.11–1.38	0.649	< 0.001

^1^ Incidence Rate Ratio

^2^ New capitation payment system,’Dental Care for Health’

^3^ Traditional Fee-for-Service payment system

## Discussion

The main finding was that the results from the 6-year follow-up were essentially repeated at the 12-year follow-up: the largest influence on the risk of increased manifest caries incidence was, still, more (≥ 3 lesions) pre-baseline caries. The impact of the payment system on the oral health risk, as defined by manifest caries, was still the second largest; however, marginally decreased from 6 to 12 years.

Over time, the distribution of educational level was altered, from being higher in FFS than in DCH after 6 years, to higher in DCH than in FFS after 12 years. This shift is more in line with earlier reports on health insurance [5, 9, 11]. However, in both models, the distribution of educational level described a more or less similar pattern, with about one third ≥ 13 years at both follow-ups. Age did not seem to have contributed to the shift.

Self-rated oral health differed between payment systems since DCH choosers scored theirs as better than FFS choosers, both after 6 and 12 years. Such a distribution would also be expected from earlier research [5, 19]. Self-reported health is considered a good predictor of future health development [20]. Health, regarded in a wider perspective, is known to describe a social gradient over a number of determinants [21], including educational level.

As a consequence, it may be reasonable to expect a covariation between the level of education and health; for example, in terms of self-assessment. However, in this study, the level of education showed covariation with self-assessed oral health in FFS but not in DCH. In other words, individuals with lower education who chose DCH did not assess their oral health as poor to a higher degree than individuals with higher education. In an earlier qualitative study, individuals who chose DCH reported “a safe habit” and “economic security” as subthemes describing attitudes towards the DCH model [22]. Scheduled, automatized payment arrangements and the recall periods determined in the contract are elements of the DCH that increase the patient´s control over the economical and timely aspects of the dental care situation. Patients are reasonably attracted by such control also by reasons that don´t relate to their level of oral health. The DCH scheme thus seem to include a compensating mechanism for the covariation of lower education with poorer health.

Caries prevalence decreased statistically significantly in both payment models since the study start and through the two follow-up periods. However, the levels were still lower in DCH than in FFS after 12 years. It is possible that increased preventative treatment may account for this decrease, at least in part. Earlier documentation from the introduction of the DCH capitation payment model indicated a larger number of preventative measures in DCH compared with FFS [9].

In a study on the oral ill health risk in FFS and DCH among young individuals in Sweden, Peterson & Twetman raised a discussion about the risk for divergent health development in the two payment models over time [23]. A free choice is likely to put the group of already resourceful individuals in a better position than those less resourceful to choose their most advantageous alternative. On the other hand, a capitation payment system might also have the capacity to provide incentives that could compensate for diverging levels of oral health. The lack of covariation of educational level and self-rated oral health in DCH but not in FFS could be interpreted in this way. Another example would be that it is reasonable to believe that the recommendation of required additional preventative treatment is more easily accepted and performed in DCH than in FFS, since such a change in the treatment plan entails no extra costs in a contractual agreement model, which it would in a per-item payment model. Earlier studies show that more preventative treatments are used in DCH than in FFS [9, 14]. It has also been suggested that a capitation payment system based on an oral health risk classification could be used as an instrument to improve society’s possibilities to direct resources; for example, to the elderly patients with the poorest health and the greatest need of support [24, 25].

To conclude, oral health in terms of manifest caries and self-reported assessment seems to differ between comparable individuals in the two payment systems with different incentives, after 6 years, and still after 12 years. The level of education seems to be related to self-rated oral health in FFS, but not in DCH. Arguably, it should be possible to take advantage of the difference in incentive structure between the payment models to improve oral health equality, instead of the opposite. At the oral health care planning level, it may be possible to use the fee class grouping based on the level of risk of oral ill health development, since efforts may be stratified according to the targeted patient category; for example, by allocating financial support according to the level of oral health, as previously suggested for the elderly, and defining a gradient of preventative care measures related to the level of oral health for younger patients.

### Strengths and limitations

The percentage of individuals retaining their 2007 payment system choice seemed to be the same in FFS as in DCH, indicating a similar, stable group of attenders in both payment models. This could be seen as an indication of a similar care panorama, and, thus, a justification for the comparison of individuals in the two payment systems, and a refutal of a substantial cluster effect. Moreover, the cohort included a substantial number of individuals essential for generalization purposes.

It is, however, important to know that these longitudinal data relate to the cohort that chose DCH (or not) in 2007. The DCH cohort of today will most likely be a different group of individuals with a different set of properties. Thus, there is a need for collection of additional data, from the present DCH and FFS groups, even though these will be cross-sectional and lack the long-term component.

The R2 measure of assessed risk for oral disease was avoided as the outcome measure due to its purpose to rather predict a future level of disease, than to give an instant measure of disease at a given time point. Instead, manifest caries incidence was chosen.

There was a difference in both presence of oral disease and risk for oral disease between the two payment system groups, as indicated by numbers of caries lesions at baseline, as well as by risk assessment level. Earlier studies on voluntary health insurances describe insurance (prepayment model) choosers as healthier than non-choosers [5]. To control for this difference, the individual number of manifest caries lesions was included also as a covariate in the regression model, as calculated pre-baseline, before the time point of payment model choice.

The results represent comparisons between the cohort that had kept their original choice of payment model after 6 years and the cohort that remained in their chosen payment scheme after 12 years. However, since all individuals in the 12-year cohort also belonged to the 6-year cohort, and the dropout rate between follow-ups was a modest 1.4% and 8.6% in DCH and FFS, respectively, the study design has been referred to as longitudinal rather than cross-sectional.

## Conclusions

The influence from the payment system on oral health development in terms of manifest caries prevalence after 12 years resembled the 6-year follow-up results, and showed remaining health differences between the payment systems. The DCH arrangements indicated a capacity to compensate for differences in health that covaried with the educational level. This may be a reason for the decision makers to consider revising the payment model, as suggested by previous research, to use the contract to allocate financial support as well as preventative care efforts according to need.

## Availability of data and material

The data sets generated and/or analyzed during the current study are not publicly available for reasons of personal and organizational integrity but are available from the corresponding author on reasonable request.

## Abbreviations

DCH: Dental Care for Health
FFS: Fee-for-Service
IRR: Incidence Rate Ratio
NDI: National Dental Insurance
PDS: Public Dental Service
VGR: Region Västra Götaland

## References

[1] Andås CA, Hakeberg M (2014). Who chooses prepaid dental care? A baseline report of a prospective observational study. BMC Oral Health.

[2] Zickert I, Jonson A, Klock B, Krasse B (2000). Disease activity and need for dental care in a capitation plan based on risk assessment. Br Dent J.

[3] Bailit HL, Newhouse J, Brook R, Duan N, Goldberg G, Hanley J, Kamberg C, Spolsky V, Black A, Lohr K. Does more generous dental insurance coverage improve oral health?, vol. 2591: Rand; 1987. 10.14219/jada.archive.1985.0407 3859541

[4] Brocklehurst P, Price J, Glenny AM, Tickle M, Birch S, Mertz E, Grytten J. The effect of different methods of remuneration on the behaviour of primary care dentists. The Cochrane Library 2013. 10.1002/14651858.CD009853.pub2 PMC6544809 24194456

[5] Doiron D, Jones G, Savage E (2008). Healthy, wealthy and insured? The role of self-assessed health in the demand for private health insurance. Health Econ.

[6] Grytten J, Holst D, Skau I (2009). Incentives and remuneration systems in dental services. Int J Health Care Finance Econ.

[7] Holloway P, Blinkhorn A, Hassall D, Mellor A, Worthington H (1997). An assessment of capitation in the General Dental Service Contract. 1. The level of caries and its treatment in regularly attending children and adolescents. Br Dent J.

[8] Holloway P, Lennon M, Mellor A, Coventry P, Worthington H (1990). The capitation study. 1. Does capitation encourage'supervised neglect'?. Br Dent J.

[9] Johansson V, Axtelius B, Soderfeldt B, Sampogna F, Lannerud M, Sondell K (2007). Financial systems' impact on dental care; a review of fee-for-service and capitation systems. Community Dent Health.

[10] Olsson C (1999). Supplier induced demand: an analysis of the Swedish dental care market.

[11] Stancil TR, Li CH, Hyman JJ, Reid BC, Reichman ME (2005). Dental insurance and clinical dental outcomes in NHANES III. J Public Health Dent.

[12] Manning WG, Bailit HL, Benjamin B, Newhouse JP (1985). The demand for dental care: evidence from a randomized trial in health insurance. J Am Dent Assoc.

[13] Andås C, Hakeberg M (2016). Payment systems and oral health in Swedish dental care: observations over six years. Community Dent Health.

[14] Andås C, Ostberg A-L, Berggren P, Hakeberg M (2013). A new dental insurance scheme—effects on the treatment provided and costs. Swed Dent J.

[15] Hallberg L, Hakeberg M, Hallberg U (2011). Facing a moral dilemma—introducing a dental care insurance within the public dental service. Swed Dent J.

[16] Ostberg A-L, Ahlström B, Hakeberg M (2012). Patients' choice of payment system in the Swedish Public Dental Service-views on dental care and oral health. Swed Dent J.

[17] Strand J, Andås A, Boman UW, Hakeberg M, Tidefors I (2015). A new capitation payment system in dentistry: the patients’ perspective. Community Dent Health.

[18] Ulfsdotter Eriksson Y, Berg K, Boman UW, Hakeberg M (2017). Contract care in dentistry: sense-making of the concept and in practice when multiple institutional logics are at play. Sociol Health Illn.

[19] Bolin K, Hedblom D, Lindgren A, Lindgren B. Asymmetric information and the demand for voluntary health insurance in Europe. In: National Bureau of Economic Research; 2010.

[20] Johnson TP (2014). Handbook of health survey methods.

[21] Marmot M (2005). Social determinants of health inequalities. The Lancet.

[22] Strand J, Andås A, Boman UW, Hakeberg M, Tidefors I (2015). A new capitation payment system in dentistry: the patients’ perspective. Community Dent Health.

[23] Petersson GH, Twetman S (2017). Relationship between risk assessment and payment models in Swedish Public Dental Service: a prospective study. BMC Oral Health.

[24] Busby M, Martin J, Matthews R, Burke F, Chapple I (2014). The relationship between oral health risk and disease status and age, and the significance for general dental practice funding by capitation. Br Dent J.

[25] Grytten J, Holst D (2013). Perspectives on providing good access to dental services for elderly people: patient selection, dentists’ responsibility and budget management. Gerodontology.

